# Attitude towards ageing and perceived health status of community-dwelling older persons in a low resource setting: a rural-urban comparison

**DOI:** 10.1186/s12877-021-02394-5

**Published:** 2021-08-06

**Authors:** E. O. Cadmus, L. A. Adebusoye, E. T. Owoaje

**Affiliations:** 1 Department of Community Medicine, College of Medicine, University of Ibadan, Oyo State Ibadan, Nigeria; 2 Chief Tony Anenih Geriatric Centre, University College Hospital, Ibadan, Nigeria

**Keywords:** Attitude towards Ageing, Older Persons, Community-Based, Nigeria

## Abstract

**Background:**

Older person’s attitude to ageing is critical for their adjustment, acceptance of health-related behaviour, survival and choices. Their attitude influences how they cope with the challenges experienced while ageing, which affects their quality of life and health-related outcomes. Despite the increasing number of older persons in sub-Saharan Africa, there is limited information about their experience. This study examines the experience and attitude of older persons in Nigeria regarding the ageing process.

**Methods:**

A descriptive cross-sectional study among older persons aged 60 years and above was carried out in a selected rural and an urban community in Oyo State, south-western Nigeria. The study participants were selected using a multi-stage sampling technique. Trained research assistants collected data with the aid of an interviewer-administered, semi-structured questionnaire. The Attitude to Ageing Questionnaire (AAQ) was used to measure participants’ perception of ageing in three domains (psychosocial loss, physical changes and psychological growth). Data were analysed using Stata version 14 at a level of significance p < 0.05.

**Results:**

A total of 1,180 participants (588 rural vs. 592 urban) were recruited for the study. The mean age was 73.2 ± 9.3 years. The majority (69.7 %) were females and still working (50.5 %). Overall, urban-dwelling participants had a better attitude to the ageing process in all the domains compared with rural-dwelling participants (psychological growth 32.5 ± 3.4 vs. 32.4 ± 3.3, *p* = 0.30; physical change 27.5 ± 5.1 vs. 26.9 ± 5.0, *p* = 0.03; and psychosocial loss 25.3 ± 5.7 vs. 25.0 ± 5.3, *p* = 0.60). Among the rural and urban-dwelling participants, good self-rated health was significantly associated with a positive attitude to ageing across the domains.

**Conclusions:**

Older persons residing in urban communities had higher positivity to ageing than their rural older counterparts. The common factor significantly associated with a positive attitude to ageing in both groups was good self-rated health. This information can be used for the planning of targeted interventions and informing policy formation for improved provisions for community-dwelling older persons in Nigeria and other sub-Saharan African countries.

## Background

Globally, many low- and middle-income countries (LMIC) are experiencing an increased growth of the older population due to improved life expectancy [1]. According to the United Nations (UN), an older person is an individual aged 60 years and above. By global estimates in 2015, older persons numbered about 900 million [1–3]. At a growth rate of about 56 % between 2015 and 2030, the United Nations estimated that older persons would increase to 1.4 billion globally. By 2050, this number is projected to grow further and will be over 2 billion [1, 4]. By 2050, about 80 % of older persons will be living in LMIC [1]. Nigeria, the most populous country in the sub-Saharan African region, is also witnessing population ageing alongside other LMIC [1]. In addition, by 2050, the number of older persons in Nigeria is expected to increase to 25.5 million from the current 6.98 million [1].

Available research shows that ageing is associated with physical, psychosocial and psychological issues. However, the ageing experience is mixed, and the perception is a continuum of positivity and negativity. [6–9]. The perception about ageing depends on how both individual and prevailing circumstances affect the individual’s attitude and coping strategies. Attitudes towards ageing are individuals’ beliefs about events, issues, or experiences [5–7] and are influenced by cultural norms, values and practices. Researchers suggest that older persons’ perspective to ageing is critical for their acceptance of health-related behaviour, survival, adjustment and choices. As such, the assessment of attitude, particularly among older persons, has been deemed beneficial [5, 8].

There are several benefits of a positive self-perceived attitude to ageing. These include physical and emotional well-being, increased life expectancy [9–12], and improved quality of life (QoL) [13–15]. Furthermore, positivity towards ageing is associated with higher resilience, personal growth, healthier outcomes [7, 11, 13, 14, 16, 17] and increased life satisfaction [6, 14, 16, 18, 19]. Conversely, older persons with a negative attitude to ageing have increased health problems and poorer health outcomes. For instance, Quin and colleagues (2009) showed that a negative attitude to ageing was associated with poor mental illness care-seeking behaviour [7]. Likewise, other research suggests that a poor attitude to ageing may be associated with depression [14, 20].

The ability to understand the experiences and attitudes of an older person regarding ageing is essential. However, studies about older persons’ attitude to ageing have to date have been mainly carried out in HIC or facility-based [6–8, 14, 21, 22]. There is, however, limited research in many LMIC [5, 12]. Typically, in low resource settings such as Nigeria, older persons reside in two distinct environments, rural and urban areas. Rural dwellers are more likely to have inadequate access to infrastructure, services and support. This inequality amplifies their vulnerabilities compared to their urban counterparts. Also, available policies do not prioritise or cater to older persons’ needs and affect their experience and attitude to ageing. The facilitation of initiatives that promote older persons to take greater responsibility for their ageing and steer members of the society towards recognising this task is a global priority [23]. There is a need to explore older persons’ perspectives about ageing and health, especially in the wake of the Decade of Healthy Ageing’s recent launch [24]. To our knowledge, this is the first study on the attitude towards ageing among older persons in Nigeria. The study set out to investigate and compare ageing attitudes among rural and urban community-dwelling older persons. Information obtained will facilitate understanding the older person’s adaptation to this phase of their existence, target negative attitudes to ageing and plan appropriate intervention and targeted policies to challenge these negative attitudes.

## Methods

This study is part of a more extensive community-based, cross-sectional study investigating the long term care (LTC) preferences of rural and urban community-dwelling older persons in south-western Nigeria [25, 26]. The study population were older men and women aged 60 years and above at their last birthday in the selected communities in Oyo State, south-western Nigeria. The sample size was determined using the sample size calculator in Stata Version 14 and the formula for comparing two proportions [27]. The assumption made for the more extensive study was a difference of 10 % between the LTC preferences of rural and urban respondents. The power was increased to 90 %, and α was set as 5 % [27]. The calculated minimum sample size in each rural and urban area was 522.

The study was carried out in Oyo State, south-western Nigeria. About 4 % of the population in the State are aged 60 years and above. Oyo State has a total of 33 Local Government Areas (LGA), of which 12 are urban, 12 are rural, while nine are peri-urban. The urban study was conducted in Ibadan North LGA, located in Ibadan, the capital city of Oyo State. From recent estimates, Ibadan North LGA has 12 wards and an estimated population of 432,900 people. Ward 3, out of the twelve wards of Ibadan North Local Government Area (LGA), was purposively selected. Based on a demographic survey conducted in 2013, Ward 3 has an estimated population of 30,861. The rural study was undertaken in Igboora, which is the headquarters of Ibarapa Central LGA. Recent estimates suggest the LGA has about 140,900 people [28]. Likewise, about 4 % of the population are aged 60 years and above.

Data were collected over two months in August and September 2018 by ten research assistants (RA) who were bilingual and had a minimum of National College of Education certificate (tertiary level of education). Two field scouts domiciled in the chosen communities facilitated community entry and identification of the selected households. Each RA was equipped with tablets for the administration of a questionnaire using the Research Electronic Data Capturing (REDCap) platform [29].

The respondents were selected through a multi-stage sampling technique to choose the LGA, wards, enumeration areas (EA) and households. The sites were purposively selected because they had already been mapped and listed by the Department of Community Medicine, College of Medicine, University of Ibadan. Initially, a rural LGA (Ibarapa Central LGA) and an urban LGA (Ibadan North LGA) were purposively selected. Next, one ward, also purposively selected, was chosen from each of the LGAs. In each of the selected wards, four EA were selected. The research assistants then visited all the households in the EA identified (from the mapping and listing database) to have individuals aged 60 years and above at their last birthdays.

A questionnaire was developed to reflect the study objectives. Information obtained included the respondent’s sociodemographic characteristics, attitude to ageing, self-rated health (SRH) and self-rated quality of Life (SRQoL). The participants were also requested to respond to the questions related to their demographic backgrounds such as age, sex, educational status, marital status, and employment. Our study assessed the factors associated with community-dwelling older persons’ attitudes using the 24 item Attitude to Ageing Questionnaire (AAQ-24) developed by Laidlaw and colleagues [16]. The tool assumes that older persons are experts in ageing and are best able to interpret their adaptation. The questionnaire measures ageing in three domains; physical functioning, psychological growth and psychosocial loss [16].

The physical changes domain assesses functioning related to the health, vitality and exercise of the individual. Together, the psychological growth and psychosocial loss domains measure the psychological experience of the older person. The psychological growth domain measures ageing gains, including coping strategies, acceptance and communication with the younger generation [16]. The psychological domain captures old age’s negative experiences, such as loss of independence, disability, loneliness and depression [16].

The perceived health status and QoL were measured using questions from the short form of measuring the QoL developed by the World Health Organization (WHOQOL-BREF) [30]. This tool has also been previously validated in Nigeria [31]. The question which assessed SRQoL was: “*How would you rate your overall quality of life?”* The responses were elicited on a five-point Likert scale ranging from (1 very bad to 5 very good). From participant responses, a dichotomous measure was created. This was coded 1 if the response was ‘very good’ or ‘good’ and 0 if the response was ‘moderate’, ‘bad’ or ‘very bad’. Having less than good (i.e., fair or poor) SRQoL was categorised as perceived poor SRQoL and those with good or better SRQoL as perceived good QoL. The question which assessed SRH was: *“In general, how would you rate your health today?”* The responses were also elicited on a five-point Likert scale ranging from (1 very bad to 5 very good). Participant responses were categorised as having good SRH (if their response to the question of SRH were “good” or “very good”. Likewise, respondents were said to have poor SRH if they reported these to be “bad”, “very bad”, or “moderate”.

The AAQ assesses three domains in the older person’s life. These are psychosocial loss, physical change, and psychological growth. The psychosocial loss domain measures both the psychological and social loss experienced by older. Higher scores in the physical change and psychological growth domain indicate positivity towards ageing [16]. The psychosocial loss domain was negatively phrased such that higher scores in the domain would indicate negativity. However, similar to previous studies, the psychosocial loss scores were reversed, in that higher scores indicate a more positive attitude [12, 32].

Researchers have validated the AAQ across different cultures and resource settings [5, 6, 21]. The instrument was translated to Yoruba, the local language and back-translated to English by a bilingual expert. After back translation, the instrument was reviewed by a panel of experts in the field of ageing. These included two community physicians, a family physician specialising in geriatric medicine, a statistician and an older person. The refined instrument was pre-tested among older persons and experts in geriatric care. The tool was pilot tested among a convenient sample of 40 participants in similar communities to those except those chosen for the study and distant enough to prevent contamination of the final study sites. The Cronbach’s alpha for the Attitude to ageing indicated good internal consistency among the items (0.76). Although Laidlaw and colleagues recommended a single total score based on a summation of all 24 items of the scale [16], this study reports within the instrument’s domains.

### Data Analysis

Data were analysed using Stata version 14 [27]. The categorical variables were summarised using proportions, while the continuous variables (including the AAQ domain scores) were summarised using means and standard deviation. Bivariate analysis to measure associations using Pearson’s chi-square test or Fisher’s exact test. The student’s t-test was used to study the statistical difference between the mean scores and dichotomous variables. Variables significant at a value of p ≤ 0.10  were entered into the linear regression model and fitted for the rural and urban locations.

## Results

Overall, 1212 older persons (600 rural versus 600 urban) were approached to participate in the study, 12 (8 urban, 4 rural) refused participation giving a total response rate of 99.0 %. The urban area’s response rate was 98.6 %, and in the rural area, 99.3 %. After uploading and initial data cleaning, 20 records were voided because the data collected were incomplete. Eventually, the data from the 1,180 completed questionnaires were analysed. These consisted of 588 (49.8 %) and 592 (50.2 %) respondents from the urban and rural respectively.

Females constituted more than half of the respondents in both locations accounting for 417 (70 %) in the urban and 406 (69.1 %) rural areas. The rural respondents were significantly older than urban respondents with a mean age of 74.2 ± 9.5 years compared to 72.3 ± 8.9 years (*p* < 0.05). The age group, 60–69 years, had the largest representation in the urban location, 248 (41.9 %), while those aged 70–79 years had the highest frequency of 221 (52.5 %) among the rural dwellers. Virtually all the respondents were of Yoruba ethnicity in rural 586 (99.7 %) and urban areas 592 (97.1 %). See Table 1.

A higher proportion of rural respondents were currently married, 281 (54.6 %), compared to the urban respondents, 234 (45.4 %). Overall, 410 (34.8 %) of the respondents reported they had some formal education. Of these, over half, 236 (57.6 %) reported they completed primary school while 88 (21.5 %) completed secondary school education. A higher proportion of respondents in the rural areas had no formal education 476 (81.0 %), compared to about half, 294 (49.7 %) of the urban respondents. Also, a higher proportion of rural respondents were still working, 325 (55.3), compared to the urban respondents, 259 (43.8 %).

**Table 1:** Socio-demographic characteristic of respondents by location

Variable	Rural *N* = 588n (%)	Urban *N* = 592n (%)	Total *N* = 1180n (%)	χ^2^	*p*-value
Sex					
Male	182 (31.0)	175 (30.0)	357 (30.3)		
Female	406 (69.0)	417 (70.0)	823 (69.7)	0.27	0.60
Age (years)					
60–69	189 (32.1)	248 (41.9)	437 (37.0)		
70–79	221 (37.6)	200 (33.8)	421 (35.7)		
80 and above	178 (30.3)	144 (44.7)	322 (27.3)	12.02	< 0.001*
Mean (SD)	74.2 (± 9.5)	72.3 (± 8.9)	73.2 (± 9.3)		
Religion					
Islam	355 (60.4)	341 ( 57.6)	696 (59.0)	1.40	0.50
Christianity	225 (38.3)	245 (41.4)	470 (39.8)		
Others	8 (1.4)	6( 1.0)	14 (1.2)		
Marital status					
Currently married	281 (54.6)	234 (45.4)	515 (43.6)		
Not currently married	307 (46.2)	358 (53.8)	665 (56.4)	8.19	< 0.001*
Formal Education					
Yes	122 (19.0)	298 (50.3)	410 (34.7)		
No	476 (81.0)	294 (49.7)	770 (65.3)	127.0	< 0.001*
Employed					
Yes	325 (55.3)	259 (43.8)	584 (49.5)		
No	263 (44.7)	333 (56.2)	596(50.5)	15.67	< 0.001*

*significant at *p* < 0.05

Table 2 shows the distribution of means and standard deviations of the AAQ domain scores by location. There were similarities in observed mean scores for the rural and urban respondents for both the psychosocial loss and the psychological growth domains. In the domain measuring physical change, urban respondents had a higher positivity towards ageing (27.5 ± 5.1) than their rural counterparts (26.9 ± 5.0). This finding was statistically significant (*p* = 0.03).

**Table 2:** Distribution of Means and standard deviations of Attitude to Ageing Questionnaire Domains scores by location

Attitude to Ageing Domains	Location	Mean (± SD)	*p*-value
**Psychosocial loss**			
	Rural	25.0 (± 5.3)	
	Urban	25.3 (± 5.7)	
	Total	25.2 (± 5.5)	0.30
**Physical Change**			
	Rural	26.9 (± 5.0)	
	Urban	27.5 (± 5.1)	
	Total	27.2 (± 5.1)	0.03*
**Psychological Growth**			
	Rural	32.4 (± 3.5)	
	Urban	32.5 (± 3.3)	
	Total	32.5 (± 3.4)	0.60

*significant at *p* < 0.05

Tables 3 and 4 show the factors associated with higher positivity in the three domains of the AAQ subscales by location. Higher positivity levels were observed among younger respondents (≤ 70 years) in the rural and urban populations in the psychosocial loss domain. Also, respondents who were married or presently in a relationship and those with formal education (*p* < 0.05) had higher positivity in the psychosocial loss domain in both locations. Likewise, respondents who were presently working and those with good SRH and SRQoL had higher positivity levels in the domain (*p* < 0.05) in both settings. There were differences in associated factors based on respondents’ location. Rural respondents living with others had higher positivity in the psychosocial loss domain than those who lived alone (*p* < 0.05). However, among the urban respondents, there were significant differences based on sex as males had higher positivity in the psychosocial domain 26.2 (± 5.6) compared to the females 25.0 (± 5.8) (*p* < 0.05).

**Table 3:** Factors associated with the subscales of the Attitude to Ageing Questionnaire (AAQ) for the Rural Population

AAQ Subscale		Psychosocial loss		Physical Change		Psychological Growth	
**Variables**	**N**	**Mean (SD)**	***p*** **value**	**Mean (SD)**	***p*** **value**	**Mean (SD)**	***p*** **value**
Age Category (in years)							
≥ 70	399	24.6 (5.5)		26.2 (5.1)		32.3 (3.6)	
≤ 70	189	26.0 (4.7)	< 0.001*	28.2 (4.7)	< 0.001*	32.8 (3.3)	0.11
Sex							
Male	182	25.3 (5.3)		27.1 (5.3)		32.5 (3.7)	
Female	406	24.9 (5.2)	0.37	26.8 (4.9)	0.49	32.4 (3.5)	0.79
Marital Status							
Currently Married	281	25.9 (4.8)		27.4 (5.0)		32.7 (3.4)	
Currently unmarried	307	24.2 (5.6)	< 0.001*	26.3 (5.0)	0.01*	32.1 (3.6)	0.03*
Formal Education							
Yes	112	26.5 (5.0)		28.8 (4.7)		33.2 (3.6)	
No	476	24.7 (5.3)	< 0.001*	26.4 (5.0)	< 0.001*	32.2 (3.5)	0.01*
Employed							
Yes	325	26.2 (4.9)		28.6 (4.3)		32.8 (3.2)	
No	263	23.6 (5.4)	< 0.001*	24.7 (5.1)	< 0.001*	31.8 (3.8)	< 0.001*
Living arrangement							
Living with others	472	25.3 (5.0)		27.2 (5.0)		32.5 (3.4)	
Living alone	116	24.0 (6.0)	0.02 *	25.7 (5.1)	0.01*	32.0 (4.0)	0.93
Home Assistance							
Yes	469	25.0 (5.2)		26.8 (5.1)		32.5 (3.5)	
No	119	25.2 (5.5)	0.73	27.3 (4.6)	< 0.001*	32.0 (3.7)	0.12
Self –rated QoL (SRQoL)							
Poor SRQoL	82	22.0 (5.5)		27.3 (4.8)		30.5 (4.0)	
Good SRQoL	506	25.5 (5.1)	< 0.001*	23.9 (5.2)	< 0.001*	32.7 (3.3)	< 0.001*
Self- rated health (SRH)							
Poor SRH	163	22.9 (5.1)		23.8 (5.2)		31.1 (3.7)	
Good SRH	425	25.8 (5.1)	< 0.001*	28.0 (4.4)	< 0.001*	32.9 (3.3)	< 0.001*

*significant at *p* < 0.05

**Table 4:** Factors associated with the subscale of the Attitude to Ageing Questionnaire (AAQ) for the Urban Population

AAQ Subscale		Psychosocial loss		Physical Change		Psychological Growth	
Variables	N	Mean (SD)	*p* value	Mean (SD)	*p* value	Mean (SD)	*p* value
Age Category							
≥ 70	344	24.5 (5.7)		26.0 (5.3)		32.3 (3.3)	
≤ 70	248	26.6 (5.6)	< 0.001*	28.2 (4.7)	< 0.001*	32.8 (3.3)	0.06
Sex							
Male	175	26.2 (5.6)		28.4 (5.3)		33.3 (3.4)	
Female	417	25.0 (5.8)	0.01*	27.1 (5.0)	1.00	32.2 (3.2)	< 0.001*
Marital Status							
Currently Married	234	27.0 (5.5)		28.7 (4.8)		33.2 (3.2)	
Currently unmarried	358	24.3 (5.7)	< 0.001*	26.7 (5.2)	< 0.001*	32.1 (3.3)	< 0.001*
Formal Education							
Yes	298	26.6 (5.6)		28.4 (5.0)		33.1 (3.2)	
No	294	24.1 (5.6)	< 0.001*	26.6 (5.0)	< 0.001*	31.9 (3.2)	< 0.001*
Employed							
Yes	259	26.5 (5.8)		28.7 (4.7)		32.8 (3.1)	
No	333	24.4 (5.5)	< 0.001*	26.6 (5.2)	< 0.001*	32.3(3.4)	0.06
Living arrangement							
Living with others	431	25.6 (5.7)		27.3 (5.2)		32.6 (3.3)	
Living alone	161	24.7 (5.8)	0.10	28.1 (4.9)	0.10	32.3 (3.3)	0.27
Home Assistance							
Yes	442	25.5 (5.7)		27.1 (5.2)		32.5 (3.3)	
No	150	24.8 (6.0)	0.16	28.7 (4.8)	< 0.001*	32.5 (3.3)	0.85
Self –rated QoL (SRQoL)							
Poor SRQoL	101	23.5 (5.6)		24.7 (5.6)		31.4 (3.5)	
Good SRQoL	491	25.7 (5.7)	< 0.001*	28.1 (4.8)	< 0.001*	32.7 (3.2)	< 0.001*
Self- rated health (SRH)							
Poor SRH	175	23.7 (5.3)		24.6 (5.4)		31.7 (3.4)	
Good SRH	417	26.0 (5.8)	< 0.001*	28.7 (4.4)	< 0.001*	32.9 (3.2)	< 0.001*

*significant at *p* < 0.05

There were similarities in factors associated with higher positivity towards physical changes in rural and urban study populations. Respondents who were younger (≤ 70years), employed, educated, married, or in a relationship had a more positive perception of physical changes. These findings were statistically significant (*p* < 0.05). Also, good SRH and good SRQoL were associated with a positive perception of physical changes in rural and urban populations. These findings were statistically significant (*p* < 0.05).

Factors associated with higher positivity in the psychological growth domain in both the rural and urban respondents were being married, educated, with good SRH and good SRQoL(*p* < 0.05). However, rural respondents who were employed were more likely to have higher positivity in the psychological growth domain compared with those who were not employed (*p* < 0.05). For urban respondents, males were more likely to have higher positivity compared to females. (*p* < 0.05).

Tables 5 and 6 show the regression analysis of the factors associated with the respondent’s attitudes towards ageing based on location. A statistically significant relationship was observed among the rural respondents for the psychosocial loss subscale of the AAQ. Positivity was observed among respondents who had a good SRH [β: 2.49 (95 % CI: 1.56–3.41)]. The regression analysis of factors associated with the respondent’s attitudes towards aspects of physical change as a result of ageing revealed that in the rural area, more positivity was seen among the educated [β: 2.03 (95 % CI:1.04–3.01)], employed [β: 3.00 (95 % CI: 2.19–3.72)] and those with perceived good self-rated health, [β: 3.50 (95 % CI: 2.70–4.30)]. Higher positivity in the psychological growth domain of the AAQ was associated with good self-rated health [β: 1.76 (95 % CI: 1.13–2.39)]

**Table 5:** Linear Regression analysis of factors associated with higher means Scores for the rural location

Domain	Psychosocial loss		Physical change		Psychological growth	
Variables	β Coefficient	95 % CI	β Coefficient	95 % CI	β Coefficient	95 % CI
Age Category						
≥ 70	-0.20	-1.16-0.76	0.45	-1.29-0.39	0.12	-0.54-0.77
< 70	1 (ref)		1 (ref)		1 (ref)	
Sex						
Male	0.59	-1.57-0.39	-0.45	-1.31-0.40	-0.34	-1.02-0.33
Female	1 (ref)		1 (ref)		1 (ref)	
Marital Status						
Currently Married	1.08	0.14–2.03*	-0.06	-0.89-0.76	0.42	-0.29-1.08
Currently unmarried	1 (ref)		1 (ref)		1 (ref)	
Living arrangement						
Living with others	0.47	-0.57-1.53	0.85	-0.06-1.77	0.28	-0.44-1.00
Living alone	1 (ref)		1 (ref)		1 (ref)	
Formal Education						
Yes	1.40	0.27–2.52*	2.03	1.04–3.01*	0.91	0.14–1.68*
No	1 (ref)		1 (ref)		1 (ref)	
Employed						
Yes	1.74	0.86–2.61*	3.00	2.19–3.72*	0.58	-0.02-1.19
No	1 (ref)		1 (ref)		1 (ref)	
Homeownership						
Owner occupied	0.45	-0.40-1.31	-0.21	-1.0-0.53	0.50	-0.02-1.12
Rented	1 (ref)		1 (ref)		1 (ref)	
Self-Rated Health						
Good SRH	2.49	1.56–3.41*	3.50	2.70–4.30*	1.76	1.13–2.39*
Poor SRH	1 (ref)		1 (ref)		1 (ref)	

* significant at *p* < 0.05

**Table 6:** Linear Regression analysis of factors associated with higher means Scores for the urban location

Domain	Psychosocial loss		Physical change		Psychological growth	
Variables	β Coefficient	95 % CI	β Coefficient	95 % CI	β Coefficient	95 % CI
Age Category						
≥ 70	-0.81	-1.80-0.17	-0.56	-1.40-0.27	-0.12	-0.70-0.46
< 70	1 (ref)		1 (ref)		1 (ref)	
Sex						
Male	-0.38	-1.57-0.81	0.15	-0.85-1.16	0.48	-0.22-1.16
Female	1 (ref)		1 (ref)		1 (ref)	
Marital Status						
Currently Married	1.56	0.42–2.69*	1.33	0.37–2.28*	0.45	-0.22-1.11
Currently unmarried	1 (ref)		1 (ref)		1 (ref)	
Living arrangement						
Living with others	0.36	-0.66-1.39	-1.15	-2.02- -0.29*	0.17	-0.43-0.77
Living alone	1 (ref)		1 (ref)		1 (ref)	
Formal Education						
Yes	1.72	0.71–2.72*	0.90	0.49–1.75*	0.70	0.10–1.29*
No	1 (ref)		1 (ref)		1 (ref)	
Employed						
Yes	1.20	0.26–2.13*	1.24	0.45–2.03*	0.24	-0.31-0.79
No	1 (ref)		1 (ref)		1 (ref)	
Homeownership						
Owner occupied	-0.43	-1.36-0.49	0.81	0.02–1.60*	0.02	-0.53-0.57
Rented	1 (ref)		1 (ref)		1 (ref)	
Self-Rated Health						
Good SRH	1.93	0.96–2.90	3.77	2.94–4.59*	1.10	0.53–1.67*
Poor SRH	1 (ref)		1 (ref)		1 (ref)	

* *p* < 0.05

In the urban community, perceived good self-rated health was significantly associated with positivity in the psychosocial loss [β:1.93 (95 % C1: 0.96–2.90)] and psychological growth domain [β:1.10 (95 %CI: 0.53–1.67)]. Factors associated with higher positivity regarding physical changes due to ageing are good SRH [β: 3.77 (95 % C1: 2.94–4.59)], employement [OR:1.24 (95 % C1: 0.45–2.03)], and marital status ([OR:1.33 (95 % C1: 0.37–2.28)].

## Discussion

This study was conducted among a sample of community-dwelling older persons in rural and urban communities in Oyo State, south-western Nigeria. The mean scores across all the domains reflected a positive attitude towards ageing based on the instrument’s authors’ recommended mean cut-off value (≥ 24) [16]. This finding is similar to a community-based study in rural Australia [14] and a facility-based study in urban New Zealand [6].

The highest positivity reported was in the domain which measured psychological growth. This finding is not surprising since this domain measures the older persons’ perceived growth in wisdom and knowledge due to ageing. Traditionally, older persons act as a repository of knowledge and understanding in the African setting. They give advice that assists in resolving family and community problems [33–35].

There were no significant differences among the rural and urban participants except in the domain which measures physical changes. A possible explanation for this finding may be because older persons face the same challenges irrespective of their setting. These challenges often result from health issues, inadequate support structures, and targeted services to cater to their needs. Research shows that older persons who require more medical attention may have to contend with a higher level of physical functioning loss [5, 7, 14]. Nevertheless, research suggests that older persons in urban locations have a more positive outlook to ageing [10]. Often, urban dwellers have higher levels of education, better job opportunities and are usually wealthier. The financial viability enables access to facilities and resources to better take care of their health and other needs [7].

There were observed rural-urban differences in factors associated with positivity across the AAQ domains. Factors associated with positivity in the psychosocial loss domain among the rural respondents were younger age, marital status and employment status. Similar to previous studies, age influences positivity in the attitude of older persons towards ageing. For instance, in a study in Malaysia, Yunus and colleagues reported that older age was significantly associated with more negative psychosocial loss scores [5]. Also, studies in Australia [14] and Edinburg [19] had similar findings. In this study, increasing age was associated with higher positivity in the physical change domain for both the rural and urban respondents and is consistent with other studies [9, 33].

Previous studies have documented sex differences in attitude to ageing [5, 6, 12, 14]. Similar to other research, this study revealed that males had higher positivity to ageing than females. This finding may be because of the inherent vulnerability of older females. Older women are less educated and less likely to have economic resources than their male counterparts [36]. Also, women usually have worse morbidities and disabilities compared to males. Furthermore, there was a significant association regarding sex in psychosocial loss and psychological growth for the urban location as males had higher positivity in the domains than females in this study. A possible explanation may be because research suggests that males gain more status with age and are regarded as more dignified than females [5].

In this study, similar to previous findings, education was associated with positivity towards ageing among rural and urban older persons [5, 10, 12, 37, 38]. Also, previous research has shown that the combination of living in urban settings and a high level of education indicate higher social status [10]. Notably, in this study, participants who were employed had more positivity in the psychosocial domain than those who were not employed [14]. Other studies have reported that older individuals with higher educational attainment have a more positive attitude towards ageing [5, 10, 39]. As Zhang and colleagues (2007) documented, educated persons are less lonely and have a higher level of psychological well-being compared to those who have lower education [39]. Also, as reported in a study among older Malaysians, education offers better occupational opportunities for financial and social status [5]. In addition, educated individuals have better resources and occupational opportunities, social and economic status [5]. Thorpe (2014) reported more negativity to physical change among older persons with low education levels [6]. Although Luo and colleagues did not use the AAQ to measure community-based older persons’ attitudes towards ageing, the study documented a similar trend [10]. According to the authors, ageing is not a uniform experience, and some people seem to fare better than others [10].

Similar to previous research, this study revealed that respondents in both locations who were presently married or in a relationship had higher positivity in the psychosocial loss and psychological growth domains [14]. This finding may be that the partners provide support, positive emotion and contribute to each other’s emotional well-being [40]. Likewise, spouses may be a source of motivation as they encourage their partners to carry out the prescribed health behaviour leading to better health outcomes [14]. Furthermore, the partner’s emotional and instrumental support may act as a buffer for stress and a direct source of positive emotion [14].

This study findings show an association between the three attitudinal domains and good self-rated health. This is similar to Yunus et al. (2015) findings in Malaysia and consistent with other studies [5, 6, 10, 19]. Similar research documented the association between self-rated satisfaction with health and higher positivity in the psychosocial loss and psychological growth domain [14]. Conversely, Bryant and colleagues (2012) revealed that increased positivity in the psychological growth domain was associated with poorer health. The authors hypothesised that this might be because individuals with poor health are more likely to generate more coping responses to their condition[14]. Also, such individuals develop more resilience [14].

Likewise, the ageing attitude has been reported to influence older persons’ physical and psychological condition and is a strong predictor of their QoL [41, 42]. For instance, a study among residents of a nursing home in Turkey indicated that a positive attitude towards ageing was associated with better QoL, satisfaction with health, lower incidence of depression and loneliness. [41]. [22]. Research suggests that individuals with higher positivity are healthier and have increased life expectancy and better life satisfaction [9, 13, 14, 43].

This study has some limitations worth mentioning. The cross-sectional nature prevents inference about the causal relationship among the factors. The study was conducted in the south-western part of the country, limiting the generalisability of the results. There is a need for future research to be conducted in other geopolitical zones of the country. Also, the study utilised quantitative measures. Further enquiries using qualitative methods to explore older people’s attitudes will be of immense benefit. Despite these limitations, this study’s findings add to the growing body of evidence on attitude to ageing in a low resource setting in SSA.

## Conclusions

This study’s added value is that, to our knowledge, this is the first study that explores the attitude of older persons in Nigeria to ageing. However, more research needs to be conducted to understand the nuances and factors that enhance positivity towards ageing in Nigeria. Identifying associated factors and predictors provides the much-needed evidence base to plan targeted intervention and policy formation. Information obtained will be beneficial and ensure older persons’ health and well-being. As shown in this study, older people at risk of a negative outlook for ageing, such as those with minimal education and those living in rural areas, must be targeted for necessary intervention.

Furthermore, the factors that will improve older persons’ lived experience, thereby improving their overall QoL, must be investigated. This is mainly because good SRH was predictive of better scores across all the domains. Other predictors such as employment emphasise the need to provide opportunities for older persons to be given opportunities to work, or proxies such as volunteering may be beneficial. Considering the effect of education on ageing attitude, opportunities to foster learning across the life span must also be explored. In terms of strengthening older persons’ physical functioning, appropriates strategies such as enabling the environment must be looked into. Social policies to promote social inclusion and other enablers of healthy ageing, such as social participation, may be beneficial regarding psychosocial loss.

## Data Availability

The datasets used and analysed during the current study are available from the corresponding author on reasonable request.

## Abbreviations

AAQ: Attitude to Ageing Questionnaire
EA: Enumeration Areas
HIC: High Income Countries
LGA: Local Government Area
LMIC: Low and Middle-Income Countries
LTC: Long tem care
QoL: Quality of life
RA: Research Assistant
REDCap: Research Electronic Data Capturing
SAGE: Study of Global Ageing and Adult Health
SRH: Self-Rated Health
SRQoL: Self-Rated Quality of Life
UN: United Nations
WHO: World Health Organization
WHOQOL-BREF: World Health Organization Quality of Life (Abbreviated)
